# The Impact of an mHealth Voice Message Service (mMitra) on Infant Care Knowledge, and Practices Among Low-Income Women in India: Findings from a Pseudo-Randomized Controlled Trial

**DOI:** 10.1007/s10995-019-02805-5

**Published:** 2019-10-04

**Authors:** Nirmala Murthy, Subhashini Chandrasekharan, Muthu Perumal Prakash, Nadi N. Kaonga, Joanne Peter, Aakash Ganju, Patricia N. Mechael

**Affiliations:** 1 Foundation for Research in Health Systems, G-1, Brigade Business Suites, 10th Main, Jayanagar 2nd Block, Bengaluru, 560011 India; 2 All of Us Research Program, National Institutes of Health, Bethesda, MD USA; 3 Present Address: BG 6011 RM 214, 6011 Exec Blvd, Rockville, MD 20852 USA; 4 Foundation for Research in Health Systems, G2, 5/26 Pillayar Kovil Street, Medavakkam, Chennai, 600100 India; 5 HealthEnabled, 6 Wherry Road, Muizenberg, Cape Town, 7945 South Africa; 6 Tufts University School of Medicine, 145 Harrison Avenue, Boston, MA 02111 USA; 7 Johnson & Johnson, 241 Main Road, Retreat, Cape Town, 7945 South Africa; 8 Mumbai, India; 9 Washington, USA

**Keywords:** mHealth, Mobile messaging, Voice messaging, Infant health, Nutrition, Digital health, Immunization, India

## Abstract

*Objectives* mHealth interventions for MNCH have been shown to improve uptake of antenatal and neonatal services in low- and middle-income countries (LMICs). However, little systematic analysis is available about their impact on infant health outcomes, such as reducing low birth weight or malnutrition among children under the age of five. The objective of this study is to determine if an age- and stage-based mobile phone voice messaging initiative for women, during pregnancy and up to 1 year after delivery, can reduce low birth weight and child malnutrition and improve women’s infant care knowledge and practices. *Methods* We conducted a pseudo-randomized controlled trial among pregnant women from urban slums and low-income areas in Mumbai, India. Pregnant women, 18 years and older, speaking Hindi or Marathi were enrolled and assigned to receive mMitra messages (intervention group N = 1516) or not (Control group N = 500). Women in the intervention group received mMitra voice messages two times per week throughout their pregnancy and until their infant turned 1 year of age. Infant’s birth weight, anthropometric data at 1 year of age, and status of immunization were obtained from Maternal Child Health (MCH) cards to assess impact on primary infant health outcomes. Women’s infant health care practices and knowledge were assessed through interviews administered immediately after women enrolled in the study (Time 1), after they delivered their babies (Time 2), and after their babies turned 1 year old (Time 3). 15 infant care practices self-reported by women (Time 3) and knowledge on ten infant care topics (Time 2) were also compared between intervention and control arms. *Results* We observed a trend for increased odds of a baby being born at or above the ideal birth weight of 2.5 kg in the intervention group compared to controls (odds ratio (OR) 1.334, 95% confidence interval (CI) 0.983–1.839, p = 0.064). The intervention group performed significantly better on two infant care practice indicators: giving the infant supplementary feeding at 6 months of age (OR 1.4, 95% CI 1.08–1.82, p = 0.009) and fully immunizing the infant as prescribed under the Government of India’s child immunization program (OR 1.531, 95% CI 1.141–2.055, p = 0.005). Women in the intervention group had increased odds of knowing that the baby should be given solid food by 6 months (OR 1.89, 95% CI 1.371–2.605, p < 0.01), that the baby needs to be given vaccines (OR 1.567, 95% CI 1.047–2.345, p = 0.028), and that the ideal birth weight is > 2.5 kg (OR 2.279, 95% CI 1.617–3.213, p < 0.01). *Conclusions for Practice* This study provides robust evidence that tailored mobile voice messages can significantly improve infant care practices and maternal knowledge that can positively impact infant child health. Furthermore, this is the first prospective study of a voice-based mHealth intervention to demonstrate a positive impact on infant birth weight, a health outcome of public health importance in many LMICs.

**Electronic supplementary material:**

The online version of this article (10.1007/s10995-019-02805-5) contains supplementary material, which is available to authorized users.

## Significance

The significance of this paper is that it is the first published study to demonstrate improved infant health outcomes and maternal health knowledge and behaviors known to improve infant health from exposure to age and stage based voice messages in a low and middle income setting, namely India.

## Introduction

Mobile health (mHealth), or the use of mobile technology in health care, is becoming an important mechanism to improve maternal, neonatal and child health (MNCH). Mobile phones enable pregnant women to receive messages to improve uptake of MNCH services that are proven to improve health outcomes. Systematic reviews assessing the effectiveness of mHealth interventions on MNCH in low- and middle-income countries (LMICs) have shown that mHealth can improve antenatal and neonatal service uptake and utilization of facility-based services. However, the reviews also recommend that more research is needed to assess impact of mHealth on clinical health outcomes (Lee et al. 2016; Sondaal et al. 2016; Chen et al. 2018). Individual studies to date have shown improvement in perinatal and neonatal mortality (Lund et al. 2012) and infant care practices, particularly related to exclusive breastfeeding (Tahir and Al-Sadat 2013; Watkins et al. 2013; Jiang et al. 2014). Recent studies have also demonstrated that mobile phone text messaging can improve the uptake of childhood vaccinations (Kazi et al. 2018) and the timely uptake of HIV PCR testing for infants for prevention of maternal HIV transmission (Coleman et al. 2017). To date there is little evidence or systematic analysis of the impact of mHealth interventions in improving infant health outcomes of birth weight or levels of malnutrition among children under the age of five, a serious public health problem in many LMICs, particularly in India (Sahu et al. 2015). Therefore, the purpose of our study was to determine if an age- and stage-based mobile phone voice messaging initiative for women, during pregnancy and up to 1 year after delivery, would lead to improved infant care knowledge and practices, and reduced levels of low birth weight and child malnutrition at 1 year of age.

## Methods

A pseudo-randomized controlled trial of the Mobile Alliance for Maternal Action (MAMA) implementation in India, called mMitra, was conducted from January 2015 to December 2017 with data collection beginning in June 2015 and ending in January 2017 (Mobile Alliance for Maternal Action Research Agenda 2015; ARMMAN, n.d.). MAMA was a four-year global initiative that aimed to improve the health and well-being of pregnant women and their newborns and infants through age- and stage-based tailored voice or text messages delivered via mobile phone (Mobile Alliance for Maternal Action Research Agenda 2015). MAMA supported the non-profit organization, Advancing Reduction in Mortality and Morbidity of Mothers, Children and Neonates (ARMMAN), to pilot a mobile messaging service and program called mMitra (ARMMAN, n.d.). The program was built on the premise that if women receive educational messages on their phone that are interesting, easy to understand, and aligned with the physiological stage of pregnancy or infant development, they will be motivated to engage in recommended self-care and seek recommended health services (Mobile Alliance for Maternal Action Research Agenda 2015). mMitra engaged pregnant women living in urban slums in Mumbai during pregnancy and through the first year of their infants’ lives. The overall aim of the program was to improve self-care and uptake of effective MNCH practices and clinical services through digital behavior change communication. The mMitra impact evaluation is registered with ISRCTN under Registration # 88968111 (See https://www.isrctn.com/ISRCTN88968111).

### Study Design and Participants

Participants were pregnant women from urban slum areas of Mumbai. Mumbai is divided into 27 municipal wards, or administrative units, each with a population of approximately 800,000–900,000 people. Each ward is typically served by one maternity home and five or six health posts that provide pregnancy and infant health services. Each ward appoints roughly 100 community health workers who make home visits, register pregnant women and motivate them to seek health care for themselves and their children. For this study, two such wards (F North and M East) were purposely selected due to their large slum area, high population proportion classified as low-income and no prior exposure to mMitra. Women speaking Hindi or Marathi language—which are spoken by over 80% of the population in the city—were enrolled in the study, and mMitra voice messages were delivered in those two languages. Women without access to a mobile phone at home or not likely to be in Mumbai for four to five months during the pregnancy and post-delivery period (i.e., those planning to visit natal homes outside Mumbai for delivery, a common cultural practice in India) were not enrolled in the study.

Pregnant women were identified and enrolled into the study by research team members. They were systematically assigned to either the intervention or control group. Group assignment was based on gestational age at the time of enrollment. For every four women enrolled consecutively, the first three were assigned to the intervention group and the last woman was assigned to the control group. The aim of the sampling was to enroll a sufficient number of women in each trimester to ensure that a dose response could be measured. The intervention group received mMitra messages; the control group did not. Women enrolled in the first trimester had a longer exposure to the messages than those enrolled in the third. All women gave their informed written consent prior to inclusion in the study. All women were followed until their infants turned 1 year of age.

### Design and Delivery of mMitra Messages

The mMitra package consisted of 145 voice messages designed to be shared from when a woman was 6 weeks pregnant until the infant reached 1 year of age. Messages were delivered two times per week during pregnancy; they were clustered at one message per day immediately post-partum for 7 days, and then reduced in frequency back to two messages per week from the second week of infancy. mMitra also provided a free call-back service within 2 days after the original call was received, in case women wanted to hear the messages they missed or listen to messages again. There were no text messages delivered through this program unlike in other programs.

The audio messages, designed by BabyCenter (BabyCenter, n.d.), were timed to the gestational age and developmental stage of the fetus and infant and based on global [World Health Organization (WHO)] and local (National Health Mission) guidelines. The messages were adapted to local practices in partnership with ARMMAN and representatives from the Federation of Obstetric and Gynecological Societies of India and Indian Academy of Pediatrics. The translations were tested for appropriateness and cultural nuances with local health experts and community focus groups. Finally, the voice and tone of the recording artist were field-tested to ensure that the messages were delivered in the reassuring tone necessary to promote the desired behavior change. The final message product was approximately 2 min in length, beginning with a recognizable ‘jingle’ to alert family members to pass a shared household phone to the pregnant woman or mother (or to place the call on speaker phone) and ending by reiterating the key element of the message. Messages were recorded in a female voice designed to represent an educated but approachable female relative.

### Sample Size and Power Calculations

The sampling procedure and sample size were determined to ensure that (a) the study population was representative of the target population and (b) the study sample size was adequate to detect a 10% reduction in the proportion of infants weighing < 2.5 kg at birth, in the intervention group as compared to the control group at an alpha of 0.05 and 80% power. The baseline for infants born with weight < 2.5 kg was determined to be 12.5% using data from the Government of India District Level Household and Facility Survey report (http://rchiips.org/DLHS-4.html). We estimated that there would be 30% attrition overall. Using z proportion test, the required sample size was estimated as 500 pregnant women per trimester in the intervention group (total n = 1500 in the intervention group) and 500 pregnant women in the control group. The intervention sample aimed to include sufficient women in the first, second, and third trimester of pregnancy to assess dose response against the same size control group which would not have any exposure to the messages and therefore were not stratified by trimester.

### Data Collection

From June to October 2015, research team members went from house-to-house in the two Mumbai wards to identify and recruit eligible pregnant women into the study. Each investigator aimed to enroll four to five pregnant women per day. This required visiting 100–110 homes per day, and 2000 women were enrolled in the study in 5 months. At the time of enrollment, the investigators administered a pregnancy survey (baseline survey) with all study participants. There were three rounds of data collection: Pregnancy (baseline/Time 1), Post-delivery (Time 2), and when the infant was 1 year old (Time 3). The survey instruments were digitized and available in Hindi and Marathi on the Kobo Collect Android-based platform. In addition to administering the surveys, the investigators also collected data from the participant’s Mother and Child Health (MCH) card, which is issued to every pregnant woman at the local health facility and updated each visit. Women are advised to retain these cards at home and bring them to every antenatal care and child health visit. The MCH cards contain information on services provided and clinical/laboratory findings (e.g., weight, BP, hemoglobin level) of the woman and the infant until the infant reaches 1 year of age.

When faced with connectivity issues or drained batteries, the investigators completed paper-based surveys and entered the data into Kobo Collect later the same day. Every evening, investigators submitted their tablets to supervisors who checked the number of completed interviews and uploaded the data onto the central server. Every day, the data manager examined the uploaded data for completeness and consistency in responses. Any problems identified were discussed in the daily morning briefings with investigators and subsequently resolved.

### Outcome Measures

The first primary outcome of interest was number of full-term infants born *at or above* the ideal birth weight of 2.5 kg. This outcome was selected as it is a marker of the baby’s health, serves as a proxy for the nutritional status of the mother throughout her pregnancy, and the data are routinely collected at birth and are available from the woman’s MCH card. The second primary outcome of interest was nutritional status at 1 year of age (Time 3). This outcome was also assessed by collecting data recorded on the MCH cards. The nutritional status of each infant was determined using weight-for-age criteria and graphing the values over time on the WHO’s z-score graph for growth monitoring.

Immunization status of the infant was assessed (at Time 3) as a secondary outcome. Being fully immunized was defined as the infant having received the schedule of vaccines under the Government of India’s Child Immunization Program, which are: one dose of Bacillus Calmette–Guérin (BCG) for tuberculosis; three doses of the pentavalent vaccine for diphtheria, pertussis, tetanus, hepatitis B and Haemophilus influenzae type B; three doses of polio and one dose of measles.

Additional outcomes focused on knowledge, attitudes and practices of women. The impact of the intervention on infant care practices was determined by comparing survey results of the final round of interviews when the infants reached 1 year of age (Time 3) in the intervention group versus control group. The changes in knowledge over time about infant care within, and across the groups (intervention, control) were also assessed by comparing the responses to ten infant care knowledge questions included in surveys, conducted during pregnancy (baseline/Time 1) and shortly after delivery (Time 2).

### Statistical Analysis

The data were analyzed using SPSS version 18.0. Descriptive analyses were conducted to ascertain the distribution of the data. Continuous data following a parametric distribution were compared between the intervention and control groups using two-paired *t* test. For the categorical outcomes, Chi square tests were conducted. Simple and binary logistic regressions were also conducted to assess the outcomes data and account for socio-demographic factors. A per protocol analysis was conducted and compared. We were unable to conduct an intention to treat analysis due to the unavailability of data. Analyses comparing the duration of exposure to the intervention were conducted by stratifying women in the intervention group by gestational age and categorizing them into three groups reflecting the total duration of exposure to mMitra by Time 3 (1–3, 4–6 and 7–9 months).

## Results

**Fig. 1:**
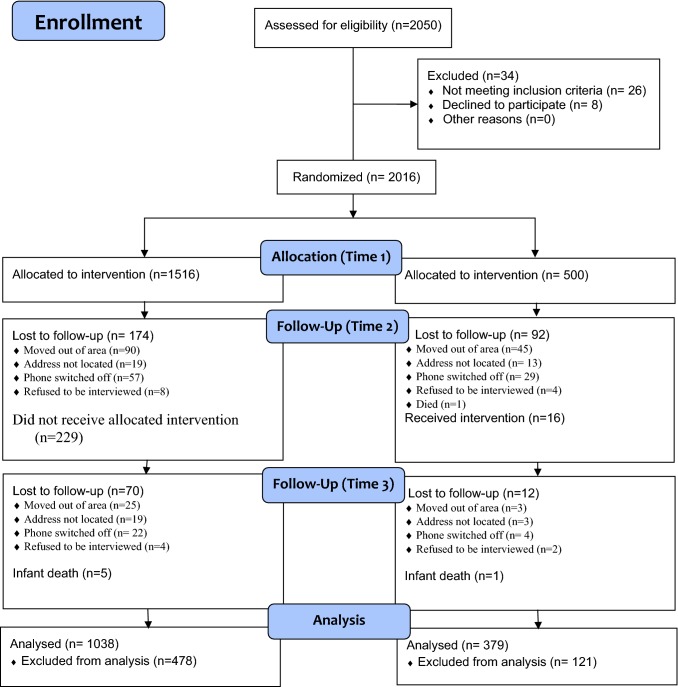
Flow diagram of women included in the mMitra pseudo-randomized control trial from Baseline through Time 3 (final follow-up period)

The mMitra impact evaluation was conducted between June 2015 and January 2017. The investigators visited over 23,500 households and identified 2050 pregnant women who spoke Hindi or Marathi as eligible. Ultimately, 2016 women were enrolled, of which 1516 were allocated to the intervention group and 500 were in the control group (Fig. 1).

### Timeline

The pregnancy (or baseline/Time 1) surveys began in June 2015 and ended in October 2015 when the requisite numbers of women were enrolled. The second round of surveys conducted shortly after the women delivered their babies (Time 2) started in November 2015 and ended in March 2016 when all of the women who were enrolled had delivered their babies. In August 2016, the third round of interviews was initiated—when babies born to women interviewed at Time 2 had started to turn 1 year old, based on the recorded delivery date—and continued until January 15, 2017.

### Characteristics of the Study Population

Of the original 2016 pregnant women enrolled, 1750 women (87%) were reached for Time 2 interviews (just after they had delivered their babies); 174 women (11.4%) were lost to follow-up in the intervention group compared to 92 (18.4%) in the control group. They had either moved out of the area, their addresses could not be located, their phones were switched off or they refused to be interviewed (Fig. 1). There was also one death in the control group. For the 1342 in the intervention group who were reachable at Time 2, 229 (17%) reported never receiving mMitra calls. Of the 408 women in the control group reachable at Time 2, 16 (3.9%) said they were receiving mMitra calls. Following per protocol analyses, the data for the women who reported never receiving the messages were discarded, reducing the post-delivery (Time 2) sample size to 1515 (intervention n = 1113; control n = 392). This was within the anticipated 30% attrition rate used in calculating the sample size.

At Time 3—when infants of enrolled women turned 1 year of age—1423 (94%) women could be contacted for follow-up: 1043 women (94%) from the intervention group and 380 women (97%) from the control group. There was greater loss to follow-up in the intervention group as compared to the control group (n = 70, 6.7% vs. n = 12, 3.1%). Six women reported infant deaths (5 in the intervention group and 1 in the control group); no information was recorded on cause of infant death in this study. The data were excluded from further analysis.

Thus, 1038 of the 1516 women originally enrolled in the intervention group (69%) and 379 of the 500 women originally enrolled in the control group (76%) were successfully followed from baseline (Time 1) up through when their infants were 1 year of age (Time 3) (Fig. 1; Table 1).

**Table 1:** Enrollment characteristics of study population in intervention and control groups by survey time point

Total enrolled (N)	Intervention			Control		
	Time 1	Time 2	Time 3	Time 1	Time 2	Time 3
	1516	1113	1038	500	402	379
Enrolled in trimester-1	260 (17.2%)	151 (13.5%)	136 (13.1%)	79 (15.8%)	55 (13.6%)	47 (12.4%)
Enrolled in trimester-2	559 (36.8%)	420 (37.7%)	387 (37.3%)	191 (38.2%)	149 (37.0%)	140 (36.9%)
Enrolled in trimester-3	697 (46.0%)	542 (48.6%)	515 (49.6%)	230 (46.0%)	198 (49.2%)	192 (50.7%)

Time 1 = baseline, Time 2 = post-delivery, Time 3 = infant at age 1 year

The women in the intervention and control groups were comparable in age, parity, education, family type, and membership in a social community (Table 2). More women in the intervention group were employed (p = 0.01).and owned a mobile phone (p = 0.0001). They were more likely to listen to the radio (p = 0.02) and read newspapers (p = 0.02). Their husbands were also more likely to be literate (0.02) and be employed (p = 0.003). Comparing Time 1 (baseline) and Time 3, for women in the intervention group, more women were likely to own a mobile phone at Time 3 compared to baseline (p = 0.008) (Online Resource 1). There were no statistically significant changes in the demographic characteristics of women in the control group at Time 1 versus Time 3.

**Table 2:** Socio-demographic characteristics of intervention and control groups at baseline (Time 1)

	Intervention N (%)	Control N (%)	p Value
Number of women	1516	500	
Variable			
Median age (years, SD)	25 (4.1)	24 (3.8)	0.361
Women’s age < 25 years	929 (61.3)	321 (64.2)	0.246
First time pregnant	462 (30.5)	165 (33.0)	0.295
Women’s education > 10 years	559 (36.9)	200 (40.0)	0.215
Woman employed	217 (14.3)	49 (9.8)	0.0099*
Living as nuclear family	803 (53.0)	250 (50.0)	0.244
Has older woman living in the house	606 (40.0)	216 (43.2)	0.207
Belong to SC/ST^a^ group	273 (18.0)	84 (16.7)	0.508
Watches TV	1264 (83.0)	401 (80.2)	0.155
Listens to radio	249 (16.4)	60 (12.0)	0.0178*
Reads newspaper	421 (27.8)	112 (22.4)	0.0176*
Woman owns mobile phone	1286 (84.8)	380 (76.0)	0.0001*
Husband literate	1339 (88.3)	421 (84.2)	0.017*
Husband employed	1490 (94.3)	488 (97.6)	0.003*

Statistically significant values are given in bold

*p < 0.05

^a^Schedule castes (SC) and schedule tribes (ST) are a set of communities identified in Indian constitution as being socially disadvantaged and therefore needing special development assistance

### Impact on Infant Health Outcomes

Of the 2016 pregnant women enrolled, 1734 women (1326/1516 of intervention group (87.5%) and 408/500(81.6%)of the control group showed their MCH cards at the time of the pregnancy baseline (Time 1) interview; 12% of women, mostly in the first trimester of pregnancy, were yet to receive MCH cards, while 19% had received the cards but did not show them to investigators. At Time 2, 1421 women, (1040/1113 (93.4%) in intervention and 381/402 (94.8%) in control group) showed their MCH cards. During this time, the data on the primary outcome of interest were collected. Subsequently at Time 3, MCH cards were available for 1046 women in the intervention group (94%) and for 378 women in control group (97%), the investigators used the updated MCH cards to abstract information on the 1-year infant health outcomes. In addition, there were similar levels of recording of babies’ birth weight and weight monitoring (i.e., weight recorded for previous 3 months) in both arms of the study (~ 65%).

For the primary infant health outcomes of interest, we detected a 33% increased odds of a baby being born at or above the ideal birth weight of 2.5 kg in the intervention group as compared to the controls; however, this finding was not statistically significant (odds ratio (OR): 1.334, 95% confidence interval (CI): 0.983–1.839, p = 0.064). At 1 year of age, there was 17% decreased odds of having a malnourished child in the intervention group as compared to controls; however, this was also not statistically significant (OR 0.823, 95% CI 0.590–1.147, p = 0.249).

We also measured a statistically significant increase in the practice of fully immunizing the infant (secondary outcome of interest) in the intervention group. Babies born to women in the intervention group had 49% increased odds of receiving all their recommended immunizations as compared to controls (OR 1.485, 95% CI 1.112–1.984, p = 0.007) as per MCH cards. However, in multivariate analysis this finding was confounded by one of the demographic variables. Babies born to women with higher “husband literacy levels” also had 55% increased odds of receiving all their recommended immunizations as compared to controls (OR 1.547, 95% CI 1.047–2.286). The impact on infants’ complete immunization status nevertheless was consistent with the self-reported rates of immunizations. Women in the intervention arm were 1.53 times more likely to report that their infant was fully immunized (OR 1.531, 95% CI 1.141–2.055, p = 0.005) (Table 3). A statistically significant difference in vaccine knowledge was also detected among women in the intervention arm compared to controls. Women in the intervention group were 1.57 times likely to know that their baby needed to be given vaccines as compared to controls (OR 1.567, 95% CI 1.047–2.345, p = 0.028) (Table 4). While there was a 10% increased odds of women in the intervention group knowing that missing a vaccine was harmful to baby, this finding was not significant (OR 1.101, 95% CI 0.837–1.449, p = 0.489) (Table 4).

**Table 3:** Impact of mMitra intervention on infant care practices after multivariable adjustment at Time 3

Practice indicator	Adjusted odds ratio (95% CI)	p value
Breastfed baby within 1 h after birth	0.86 (0.67–1.1)	0.23
Women fed colostrum to babies	1.29 (0.86–1.94)	0.20
Babies not given honey etc. in the first 3 days	0.9 (0.69–1.16)	0.42
Babies had health checkup at hospital discharge	1.11 (0.86–1.43)	0.40
Baby was weighed at least once in previous 3 months	0.77 (0.6–0.98)	0.03
Baby was breastfed for 6 months or more	0.82 (0.48–1.4)	0.48
Baby was given supplementary feeding at 6 months	**1.4 (1.08–1.82)**	0.009**
Specific food items baby ate the previous day:		
Rice/chapati/bread	0.96 (0.54–1.69)	0.89
Pulses and lentils	0.88 (0.69–1.12)	0.30
Vegetables	0.84 (0.65–1.1)	0.21
Fruits	1 (0.76–1.32)	0.97
For infants having had diarrhea	0.94 (0.71–1.24)	0.68
Took ORS	0.96 (0.56–1.65)	0.90
Took ORS + Zinc	1.24 (0.67–2.31)	0.48
Continued to feed baby during diarrhea	0.88 (0.34–2.3)	0.80
Infant fully immunized^a^	**1.531 (1.141–2.055)**	0.005**

*p < 0.05, **p < 0.01

^a^Full immunization schedule was considered as one dose of BCG, three doses of Pentavalent, three doses of polio, and one dose of measles (as prescribed under the Government of India’s child immunization program)

**Table 4:** Impact of mMitra intervention on infant care knowledge after multivariable adjustment at Time 2

Knowledge indicator	Adjusted odds ratio (95% CI)	p value
Newborn baby should not be given honey	1.269 (0.977–1.647)	0.073
Newborn should be breastfed within 1 h	1.4 (0.978–2.006)	0.065
Baby not able to suckle, should be given outside milk	0.597 (0.272–1.31)	0.198
Newborn baby should not be given water	0.897 (0.697–1.153)	0.396
Baby should be given solid food by age 6 month	**1.89 (1.371–2.605)**	< 0.01***
Ideal birth weight of a baby is > 2.5 kg	**2.279 (1.617–3.213)**	< 0.01***
Baby needs to be given vaccines	**1.567 (1.047–2.345)**	0.028**
Mother knew missing any vaccine is harmful to baby	1.101 (0.837–1.449)	0.489
Feeding baby during diarrhea, does not aggravate diarrhea	1.08 (0.813–1.434)	0.593
Do you think, baby needs to be weighed periodically	0.934 (0.651–1.34)	0.713

Statistically significant values are given in bold

Outcomes reflect proportion of women in each group providing correct answers at Time 3

**p < 0.05; ***p < 0.01

### Impact on Infant Care Practices

For the 15 practice indicators, both the control and intervention group recorded high achievement overall. When the two groups were compared to assess statistically different changes in infant care practices, the intervention group performed significantly better on two practice indicators as compared to the control group, which were: giving the infant supplementary feeding at 6 months of age (OR 1.4, 95% CI 1.08–1.82, p = 0.009) and fully immunizing the infant (see previous section) (Table 3). In addition, women in the intervention group tended to perform better than controls for feeding colostrum to babies, ensuring baby had a health checkup at discharge, and giving their infant oral rehydration solution (ORS) plus zinc during diarrheal episodes. However, these were not statistically significant (p = 0.2 and 0.4, respectively).

By contrast, the control group performed better than intervention group on the following practices: breastfeeding the baby within 1 h of birth; having the baby weighed within 3 months; breastfeeding the baby for a minimum of 6 months; diversifying diet with pulses, lentils and vegetables; and continuing to feed their infant during episodes of diarrhea. Only differences in having the baby weighed within 3 months were significantly increased in the control group (p = 0.03 OR 0.77, 95% CI 0.6–0.98).The groups performed similarly for the remaining practices, including not giving the baby honey within 3 days of birth; feeding their infant rice, chapati, bread and fruits; and giving ORS (only) during diarrhea.

### Impact on Infant Care Knowledge

For three of the ten knowledge indicators, the intervention group was statistically significantly different than the control group (Table 4). Women in the intervention group were at increased odds of knowing that a baby should be given solid food by 6 months (OR 1.89, 95% CI 1.371–2.605, p < 0.01), that a baby needs to be given vaccines (see “Impact on Infant Health Outcomes” section), and that the ideal birth weight is > 2.5 kg (OR 2.279, 95% CI 1.617–3.213, p < 0.01).We measured a trend towards increased knowledge in the intervention group compared to controls for the following topics: baby should not be given honey and that the baby should be breastfed. Conversely, women in the control group showed increased knowledge in supplementing baby’s food if baby was unable to suckle and not giving a newborn water. These changes were not statistically significant (Table 4). The two groups were similar in knowledge on diarrhea management, the need for weighing the baby periodically and that missing any vaccines was harmful (Table 4).

### Impact of mMitra Exposure

There were differences observed on select practice and knowledge indicators based on length of exposure to mMitra. Women receiving messages for 7–9 months performed significantly better compared to women receiving messages for 1–3 months on five infant care practices, especially in two key immediate infant care practices: breastfeeding the baby within an hour after birth and not feeding honey to the baby in the first 3 days (Online Resource 2). There were also significant improvements in these two practices in women who received mMitra messages for 4–6 months as compared to 1–3 months.

There were also significant improvements in certain types of nutritious foods included in the infant’s diet with longer periods of exposure to mMitra (7–9 months compared to fewer than 6 months); however, there was no significant difference between the control and intervention groups overall in these practices. In contrast, although there was a significant increased difference in self-reporting that the baby was fully immunized among women receiving the mMitra intervention as compared to controls, there was no detection of a dose response.

Similar analyses for knowledge indicators showed that women receiving mMitra for 7–9 months had significantly higher levels of knowledge on four of the topics as compared to women exposed for fewer than 6 months of messages (Online Resource 3). These overlapped with the practice indicators.

## Discussion

Our study findings provide robust evidence, obtained through a pseudo-randomized controlled trial, that tailored mobile phone voice messages can improve key infant care knowledge and practices that lead to improved infant health outcomes in low-resource settings. The findings align with other studies that have reported positive impact of text message reminders on uptake of neonatal services for immunization and completion of the immunization schedule within the first year of birth (Sondaal et al. 2016). Future research could assess whether voice messages may be more effective in promoting uptake of immunization compared to text messages in some populations or can further increase uptake of immunization in populations where baseline coverage is high but stagnated, to achieve goals of complete childhood immunization (World Health Organization 2018). Such research could also examine if increasing the length of exposure to mobile messages could increase uptake and completion of infant immunization in high baseline settings.

In this study, we specifically attempted to assess direct impact on primary infant health outcomes, normal infant birth weight and reduction of malnutrition, by using clinical anthropometric data about the infant (weight and height), recorded in the MCH cards. We show that tailored voice messages can improve odds of a baby being born at, or above the ideal birth weight of 2.5 kg.

Although we had access to MCH cards of ~ 94% of women in both intervention and control groups, we found only around 65% cards contained data on weight measurements within the last 3 months. However, we found no difference in the levels of weight recording in the last 3 months between the groups. Although, overall, numbers of infants at the appropriate nutritional level remained low in both arms (around 35%), the small increased proportion that we detected among women receiving the mMitra messages is promising, especially given the significant increase in self-reported supplementary feeding practices among women receiving the mMitra intervention. Education about complementary feeding has been shown to improve growth and development among children in India (Vazir et al. 2013) and there is limited but mixed evidence of the impact of complementary feeding interventions on stunting (Dewey 2016; Bhutta et al. 2013). As a recent systematic review shows, information, education and counselling interventions have a small but significant impact on height and linear growth of infants in low- and middle- income countries (Panjwani and Heidkamp 2017). Future research could explore how voice-based mobile messages compare to standard education methods and whether combining mobile voice messaging with complementary food interventions can reduce stunting and increase infant height and weight in low-resource settings.

While we did detect a significant increase in knowledge among women in the intervention arm about the ideal birth weight of the baby, and a slight trend for increasing knowledge on this topic with longer exposure to mMitra, we did not observe a significant impact of mMitra on infant birth weight. This finding is unsurprising given that birth weight of the infant may depend on several factors including the diet, nutritional status, level of rest, and pregnancy complications among the women. Also, to date few mHealth interventions, if any, have systematically assessed impact on infant birth weight or demonstrated a positive effect on improving birth weight. Only one study from South Africa recently reported lower risk of delivering a low birth weight infant among women receiving text messages, although there was no significant difference between mean infant birth weight compared to controls (Coleman et al. 2017). Ongoing work in eight African countries as part of the GSMA mNutrition initiative (GSMA, n.d.) has shown increase in knowledge on infant care practices such as initiation of breastfeeding within one hour of birth, exclusive breastfeeding, appropriate food supplementation with vitamins and minerals and adherence to appropriate nutrition practices among users of the mHealth interventions compared to non-users. However, it remains to be seen if these interventions will assess impact on child health outcomes such as birth weight, stunting and malnutrition (GSMA, n.d.).

Women exposed to mMitra messages made significant improvements in a key infant practice, supplementary solid feeding of the baby at 6 months of age, that potentially improves the growth and nutritional status of the infant. We also observed a statistically significant difference in knowledge on the related topic between the intervention and control groups. Interestingly, we did detect a significant trend for improving levels of knowledge about this topic with greater exposure to mMitra among women in the intervention group. We did not detect an overall difference between women exposed to mMitra compared to controls in the specific types of food they included to supplement their infant’s diets. However, we did observe a dose-dependent increase in the practice of diet supplementation among women in the intervention group. While it is reasonable to assume that changes in knowledge about infant care would also correspond with changes in infant care practices, knowledge indicators measured in this study were not directly correlated to specific practice indicators.

There was a discrepancy between the knowledge of breastfeeding within the first hour of birth and practice. The intervention group had a small decrease in practice but increased knowledge about the practice. One potential explanation for this inconsistency could be that a woman’s ability to undertake immediate breastfeeding is influenced by factors such as type of delivery (vaginal/cesarean), complications during delivery and hospital protocols, for which we do not have information. We also observed a significant increase in knowledge on breastfeeding within first hour of birth, and the practices of breastfeeding within the first hour of birth and feeding colostrum among women receiving mMitra intervention for longer periods of time. These trends suggest that extending the period of mobile messaging through the entire pregnancy and not just the third trimester may complement, and even strengthen, traditional educational strategies to increase feeding colostrum and breastfeeding within the first hour of birth even in contexts of high levels of familiarity with these practices. These trends are also in line with findings of recent systematic reviews that showed text messages compared to routine care can improve rates of breastfeeding within an hour of birth (Lee et al. 2016; Sondaal et al. 2016). However, in the meta-analysis they conducted, Lee et al. found poor evidence of mHealth interventions improving feeding of colostrum to the baby among women exposed to mobile messages (Lee et al. 2016). Further research is necessary to systematically assess whether and how the dosage and timing of messaging, along with the modality of such messages (voice vs. text), can improve initiation of breastfeeding and feeding colostrum.

### Limitations

Our study had several limitations. First, the way the study participants were randomized is error prone. There is a possibility that the protocol for group assignment was not adhered to; it could also lead to an unbalanced sample. It was also recognized that we would have challenges finding enough women in the first trimester to reach the 500-women sample size target, hence we instead focused on an overall sample size of 1500 women in the intervention group. Secondly, there was a high proportion of attrition in both arms, which could lead to selection bias. At baseline (Time 1), all women in the intervention group were to receive the intervention. At Time 2, we discovered women in the intervention group who did not receive the messages and women in the control group who indicated that they did receive the messages. Those who said they were receiving the messages in the control group could have self-enrolled in mMitra as city-wide campaigns for the initiative began after the research study had begun. These shifts in exposure led to the inability to conduct intention to treat analysis as it was impossible to determine who was exposed to what messages and link to associated outcomes, leading to an as treated approach to the analysis. At Times 2 and 3 the number of those who “moved out” was large as expected because in slums, people are expected to move from one house to another when their eleven-month leases expire. In addition, there were women for whom we had some addresses that could not be located because there were no recognizable landmarks or landmarks were not correctly noted. In all cases, investigators first tried to reach all potential losses to follow up by phone. In cases of phones switched off or not responded to, they made three attempts to call back at different times. If the phone was still switched off, they were considered as lost to follow-up. If women could be reached by phone and the new locations were far off from Mumbai, then those women were marked as “moved out of area”.

While triangulation was attempted between the MCH cards and the surveys, both sources were limited. The MCH cards were the data source for the primary outcomes; however, only 65% of the available cards in both groups had the necessary anthropometric data. It is unclear how the lack of that data affected the statistical outcome. We also do not know what delivery complications took place and if there were differences in delivery complication rates between the two groups, which could have influenced some of the outcomes (clinical and practice). The surveys relied on self-reporting, which potentially introduced biases such as recall and social desirability biases. Another limitation is that the intervention messages did not map to specific indicators with multiple messages supporting more than one health outcome and single messages supporting multiple outcomes. Therefore, it was not possible to perform a one for one correlation between the messages and the outcomes. Lastly, while infant care knowledge seemed to increase with exposure to mMitra, it is not possible to fully attribute any changes to the intervention alone due to temporal trends of increased knowledge observed in both control and intervention groups. This trend is not entirely surprising, as all women were expected to have access to other sources of similar information, including community health workers who enroll women for pregnancy care; health care providers; and mass media messages on radio, TV, posters, etc.

## Conclusion

This study adds to the growing body of evidence on the impact of mHealth interventions with statistically significant differences in several infant care practices and a dose response effect on knowledge and behaviors known to improve neonatal and infant health outcomes. To our knowledge, this is one of the first voice message interventions for MNCH to be evaluated systematically by a prospective experimental design in a low-resource setting. Furthermore, this study may be the first *prospective* mHealth study to demonstrate a positive impact on an important infant health outcome, infant birth weight. Further research is recommended to assess the relationship between changes in knowledge and behavior. Additional studies should also compare voice versus text message interventions on MNCH outcomes. Such research should also systematically explore the differential impacts of tailored voice messages compared to text messages and align them with the specific behavior/practice changes of interest.

## Electronic supplementary material

Below is the link to the electronic supplementary material.

Supplementary material 1 (DOCX 27 kb)
