# Supplementary material for: The Impact of an mHealth Voice Message Service (mMitra) on Infant Care Knowledge, and Practices Among Low-Income Women in India: Findings from a Pseudo-Randomized Controlled Trial
Source: Matern Child Health J. 2019 Oct 4;23(12):1658–69. doi: 10.1007/s10995-019-02805-5 (PMC6823296; doi:10.1007/s10995-019-02805-5)
Supplement: Supplementary file 1 — Supplementary material 1 (DOCX 27 kb) [file 10995_2019_2805_MOESM1_ESM.docx]

**Online Resource 1 Comparison of socio-demographic characteristics of intervention and control group participants at Time 1 and Time 3**

| **Variable** | **Intervention (in R1)** | **Intervention (in R3)** | **p value (R3-R1)** | **Control (in R1)** | **Control (in R3)** | **p value** |
| --- | --- | --- | --- | --- | --- | --- |
| **Number of women** | 1516 | 1038 |  | 500 | 379 |  |
| Median age of women | 25 (SD 4.1) | 25 (SD 3.8) | 0.489 | 24(SD 3.8) | 24 (SD 4.0) | 0.971 |
| 1.Women's age < 25 years | 61.3 | 59.8 | 0.446 | 64.2 | 64.3 | 0.976 |
| 2. First time pregnant | 30.5 | 29.7 | 0.665 | 33.0 | 31.5 | 0.638 |
| 3. Women's education - 10+ years | 36.9 | 39.9 | 0.125 | 40.0 | 42.6 | 0.438 |
| 4. Woman employed | 14.3 | 15.5 | 0.401 | 9.8 | 10.0 | 0.922 |
| 5. Living as Nuclear family | 53.0 | 50.3 | 0.179 | 50.0 | 48.9 | 0.746 |
| 6. Has older woman living in the house | 40.0 | 41.7 | 0.390 | 43.2 | 43.4 | 0.953 |
| 7. Belong to SC/ ST ^1^group | 18.0 | 17.8 | 0.897 | 16.7 | 17.2 | 0.845 |
| 8. Watches TV | 83.4 | 86.1 | 0.064 | 80.2 | 79.9 | 0.854 |
| 9. Listens to radio | 16.4 | 15.3 | 0.456 | 12.0 | 11.1 | 0.68 |
| 10. Woman owns mobile phone | 84.8 | 88.5 | **0.008*** | 76.0 | 81.0 | 0.076 |

*p<0.05, ^1^Schedule castes (SC) and Schedule Tribes (ST) are a set of communities identified in Indian constitution as being socially disadvantaged and therefore needing special development assistance.

**Online Resource 2 Impact of length of exposure to mMitra messages on infant care practices**

| **Infant care Practice Indicators** | **7 - 9 months**  **(A)** | **4 - 6 months**  **(B)** | **1-3 months**  **(C)** | **B-C** | | | **A-B** | | | **A-C** | | |
| --- | --- | --- | --- | --- | --- | --- | --- | --- | --- | --- | --- | --- |
| **N** | n= 136 | n= 387 | n= 515 | Diff | p value | 95% CI | Diff | p value | 95% CI | Diff | p value | 95% CI |
| 1.Breastfed baby within one hour after birth | 73.1 | 53.1 | 46.3 | 6.8 | **0.022**** | 1.27-12.33 | 20 | **0.0001***** | 11.92-28.08 | 26.8 | **0.0001***** | 18.88-34.72 |
| 2. Women fed colostrum to babies | 97 | 92.1 | 91.4 | 0.7 | 0.353 | -2.35-3.75 | 4.9 | **0.024**** | 0.82-8.98 | 5.6 | **0.013**** | 1.44-9.76 |
| 3. Babies not given honey, water etc in the first 3 days | 79.9 | 67.3 | 60.2 | 7.1 | **0.014**** | 1.76-12.44 | 12.6 | **0.003**** | 5.13-20.07 | 19.7 | **0.0001***** | 12.1-27.3 |
| 4. Babies had health checkup at hospital discharge | 71.3 | 64.6 | 70.5 | -5.9 | 0.031** | 0.74-11.06 | 6.7 | 0.078 | -1.05-14.45 | 0.8 | 0.427 | -6.42-8.02 |
| 5. Baby was weighed at least once in previous 3 months | 95.6 | 97.4 | 97.4 | 0.0 | 0.5 | -1.76-1.76 | -1.8 | 0.148 | -1.03-4.63 | -1.8 | 0.136 | -0.89-4.49 |
| 6. Baby was breastfed for 6 months or more | 91.2 | 84.8 | 90.7 | -5.9 | **0.003***** | 02.33 – 9.47 | 6.4 | 0**.030**** | 0.79 – 12.01 | 0.5 | 0.428 | -4.08 - 5.08 |
| 7. Baby was given supplementary feeding at 6 months | 73.5 | 65.4 | 67 | -1.6 | 0.307 | -3.63-6.83 | 8.1 | **0.041**** | 0.42-15.78 | 6.5 | 0.074 | -0.88-13.88 |
| **Specific food items baby ate yesterday** |  |  |  |  |  |  |  |  |  |  |  |  |
| 8. Rice/Chapati/bread | 89 | 97.2 | 94.8 | 2.4 | 0.037** | 0.19-4.61 | -8.2 | 0.0001*** | 4.65-11.75 | -5.8 | 0.007*** | 1.92-9.68 |
| 9. Pulses and lentils | 36.8 | 47.9 | 51.5 | -3.6 | 0.142 | -1.93-9.13 | -11.1 | 0.013** | 2.94-19.26 | -14.7 | 0.001*** | 6.77-22.63 |
| 10. Vegetables | 24.3 | 30.8 | 28.5 |  | 0.227 | -2.75-7.35 | -6.5 | 0.076 | -0.95-13.95 | -4.2 | 0.165 | -2.89-11.29 |
| 11. Fruits | 16.9 | 28.5 | 24.3 | 2.3 | 0.077 | -0.66-9.06 | -11.6 | 0.004*** | 4.45-18.75 | -7.4 | 0.034** | 0.75-14.05 |
| 12. Child Fully Immunized | 82.4 | 79.3 | 83.1 | -3.8 | 0.073 | -0.5 - 08.1 | 3.1 | 0.218 | -3.45 - 9.65 | -0.7 | 0.423 | -5.26 - 6.66 |
| **Number of Child suffered from** diarrhea | 31 | 105 | 112 |  |  |  |  |  |  |  |  |  |
| 13. Took ORS | 74.2 | 70.5 | 67.9 | 2.6 | 0.339 | -7.72-12.92 | 3.7 | 0.344 | -11.5-18.9 | 6.3 | 0.251 | -09.1-21.7 |
| 14. Took ORS + Zinc | 16.1 | 24.8 | 20.5 | 4.3 | 0.225 | -5.04-13.64 | -8.7 | 0.155 | -5.41-22.81 | -4.4 | 0.292 | -8.84-17.64 |
| 15. Continued to feed baby during diarrhea | 93.5 | 92.4 | 94.6 | -2.2 | 0.255 | -3.29-7.69 | 1.1 | 0.418 | -7.67-9.87 | -1.1 | 0.407 | -6.6-8.8 |

** p< 0.05, ***p<0.01

**Online Resource 3 Impact of length of exposure to mMitra messages on infant care knowledge**

| **Knowledge Item** | **7 - 9 months (A)** | **4 - 6 months**  **(B)** | **1-3 months**  **(C)** | **B-C** | | | **A-B** | | | **A-C** | | |
| --- | --- | --- | --- | --- | --- | --- | --- | --- | --- | --- | --- | --- |
| **N** | 136 | 387 | 515 | Diff | P value | 95% CI | Diff | p value | 95% CI | Diff | P value | 95% CI |
| 1. Ideal birth weight of a baby is >2.5kg | 94.9 | 88.4 | 91.5 | -3.1 | 0.061 | -019-6.39 | 6.2 | **0.015**** | 1.6-11.4 | 3.4 | 0.094 | -0.85-7.65 |
| 2.Newborn baby should not be given honey etc* | 89 | 78.6 | 73 | 5.6 | **0.026**** | 0.83-10.37 | 10.4 | **0.004***** | 4.01-16.79 | 16 | **0.0001***** | 9.26-22.74 |
| 3. Newborn should be breastfed within one hour | 96.3 | 90.4 | 87 | 3.4 | 0.057 | -0.14-6.94 | 5.9 | **0.015**** | 1.44-10.36 | 9.3 | **0.001***** | 4.33-14.27 |
| 4. Baby not able to suckle, should be given outside milk | 93.4 | 96.6 | 97.3 | -0.7 | 0.271 | -1.19-2.59 | -3.2 | 0.055 | -0.1-6.5 | -3.9 | 0.014 | 0.98-6.82 |
| 5. Newborn baby should not be given water | 89.7 | 85.5 | 81 | 4.5 | **0.037**** | 0.34-8.66 | 4.2 | 0.108 | -1.39-9.79 | 8.7 | **0.008***** | 2.72-14.68 |
| 6. Baby should be given solid food by age 6 month | 94.9 | 86.6 | 88 | -1.4 | 0.265 | -2.27-5.07 | 12.5 | **0.0042***** | 3.12-13.48 | 6.9 | **0.010**** | 2.03-11.77 |
| 7. Baby needs to be given vaccines | 95.6 | 91.5 | 92.2 | -0.7 | 0.352 | -2.32-3.72 | 4.1 | 0.058 | -0.2-8.4 | 4.1 | 0.085 | -0.67-7.47 |
| 8. Mother knew, missing any vaccine is harmful to baby | 83.1 | 86.8 | 86.6 | 0.2 | 0.465 | -3.56-3.96 | -3.7 | 0.1435 | -2.02-9.42 | -3.5 | 0.148 | -2.02-9.02 |
| 9. Feeding baby during diarrhea, does not aggravate diarrhea | 11.8 | 31.8 | 35.7 | -3.9 | 0.111 | -1.34-9.14 | -20 | 0.0001 | 012.76-27.24 | -23.9 | 0.0001 | 16.59-31.21 |
| 10. Baby needs to be weighed periodically | 91.2 | 86.8 | 86.6 | 0.2 | 0.465 | -3.56-3.96 | 4.4 | 0.087 | -0.94-9.74 | 4.6 | 0.074 | -0.63-9.83 |

** p< 0.05, ***p<0.01
